# ABOT: an open-source online benchmarking tool for machine learning-based artefact detection and removal methods from neuronal signals

**DOI:** 10.1186/s40708-022-00167-3

**Published:** 2022-09-01

**Authors:** Marcos Fabietti, Mufti Mahmud, Ahmad Lotfi, M. Shamim Kaiser

**Affiliations:** 1 Department of Computer Science, Nottingham Trent University, Clifton Lane, Nottingham, NG11 8NS UK; 2 Medical Technologies Innovation Facility, Nottingham Trent University, Clifton Lane, Nottingham, NG11 8NS UK; 3 Computing and Informatics Research Centre, Nottingham Trent University, Clifton Lane, Nottingham, NG11 8NS UK; 4 Institute of Information Technology, Jahangirnagar University, Dhaka, 1342 Savar, Bangladesh

**Keywords:** Computational neuroscience, Neuronal spikes, Local field potentials, Electrocorticogram, Electroencephalogram, Magnetoencephalogram

## Abstract

Brain signals are recorded using different techniques to aid an accurate understanding of brain function and to treat its disorders. Untargeted internal and external sources contaminate the acquired signals during the recording process. Often termed as artefacts, these contaminations cause serious hindrances in decoding the recorded signals; hence, they must be removed to facilitate unbiased decision-making for a given investigation. Due to the complex and elusive manifestation of artefacts in neuronal signals, computational techniques serve as powerful tools for their detection and removal. Machine learning (ML) based methods have been successfully applied in this task. Due to ML’s popularity, many articles are published every year, making it challenging to find, compare and select the most appropriate method for a given experiment. To this end, this paper presents ABOT (Artefact removal Benchmarking Online Tool) as an online benchmarking tool which allows users to compare existing ML-driven artefact detection and removal methods from the literature. The characteristics and related information about the existing methods have been compiled as a knowledgebase (KB) and presented through a user-friendly interface with interactive plots and tables for users to search it using several criteria. Key characteristics extracted from over 120 articles from the literature have been used in the KB to help compare the specific ML models. To comply with the FAIR (Findable, Accessible, Interoperable and Reusable) principle, the source code and documentation of the toolbox have been made available via an open-access repository.

## Introduction

Neuronal signals are one of the cornerstones of neuroscience in understanding brain activity. They can be acquired non-invasively with methods such as electroencephalography (EEG) and magnetoencephalography (MEG), as well as invasively in the cases of electrocorticography (ECoG), local field potentials (LFP), and neuronal spikes [1, 2]. They are crucial in diagnosing and treating brain disorders, including neurodegenerative diseases and mental health problems. These include Alzheimer's disease, cognitive impairments, schizophrenia, Parkinson's disease, dementia, epilepsy, migraine, and sleep disorders, to name a few [3]. Additionally, they are used for establishing brain–computer interface (BCI) applications targeting rehabilitation and restoring motor functionality [4–8].

**Fig. 1:**
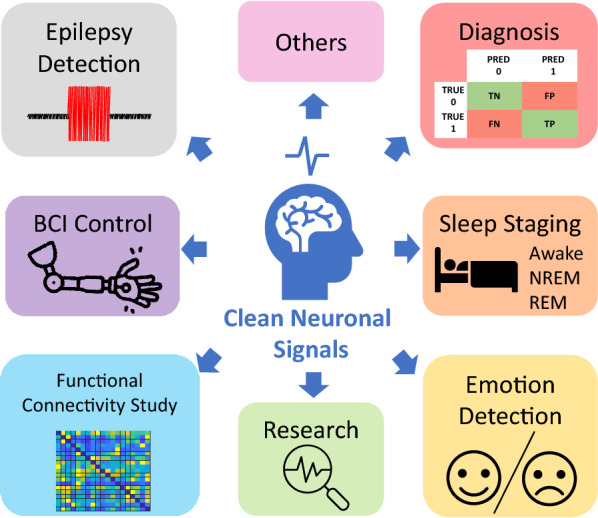
Overview of the application areas that benefit from the removal of artefacts

However, acquiring the neuronal networks' activities requires sophisticated electrical and mechanical apparatus within the proximity of the sensors. Moreover, the acquired neuronal activities can also be transferred using wired or wireless interfaces for digitisation and storage. All this equipment used in the process introduces unavoidable contamination to the acquired signal [9–11]. This contamination, popularly known as an artefact, can be physiological or caused by an external source [12]. The undesired effects of its present range from causing a BCI device to operate erroneously, misdiagnosis of diseases or brain conditions (as in the diagnosis of schizophrenia, sleep disorders and Alzheimer's disease [13]) or producing false alarms (as in generating false alarms for brain seizures [14]). A collection of applications that benefit from artefact removal, such as the aforementioned ones, is presented in Fig. 1.

Given the consequences of artefacts on acquired neuronal signals, many scientists have been interested in developing methods for detecting and removing them [15]. Several approaches are proposed for this, for example, filtering out the specific spectrum of an artefact. However, several artefacts have a broad frequency band and cannot be easily filtered [9]. Another known strategy is an expert's manual review of the neuronal recording and discarding of contaminated segments. Nonetheless, the information loss is significant and highly undesirable [16]. Because of this, different automatic techniques have been developed to classify and remove artefacts to preserve the information. These include blind source separation, wavelet decomposition, regression, empirical-mode decomposition, template subtraction, adaptive filtering, and other variations or hybrid approaches, each with pros and cons [15, 17].

However, there has been a rise in recent years of new approaches based on machine learning (ML) techniques [26], as there are benefits of employing them over other computational methods. First, their inherent characteristic for not requiring expert observation to classify artefacts, as they recognise the patterns in the data, improves the classification accuracy. Consequently, ML techniques outperform other methods in terms of classification performance [19]. In addition, they do not require a reference channel, unlike regression or filtering methods [15]. They can be used in single-channel recordings, unlike independent component analysis (ICA), which needs the number of recording channels equal to the number of independent sources [21]. Moreover, they are more flexible than template subtraction, which may add new artefacts due to the inaccuracy of the reconstructed template, as both the artefacts and the signals have complicated shapes. They can also remove artefacts that overlap in the spectral domain, unlike wavelet decomposition, which may cause information loss and faulty reconstruction of clean signals [16]. Lastly, they are computationally efficient, allowing for online applications boosted by hardware accelerators [27].

The extensive literature on ML-based solutions can be overwhelming to find, compare and select the best method which suits the researcher's experiments. This is aggravated for the experimenters who do not need to know the technical details of the artefact detection and removal process to select an appropriate method for their acquired signals [28, 29]. In the literature, we find meta-studies of artefact detection and removal methods that are compiled in Table 1. It is shown that not all of the reviews describe the various artefacts, and that all of them focus exclusively on EEG and not the other neuronal signals. Furthermore, only four of them mention ML-based methods, with only a small subset of the available literature compared. Overall, there exists a lack of a holistic overview of the artefact detection and removal across all neuronal signals from an ML perspective.

**Table 1:** Comparison of available reviews on methods applied to artefact removal, sorted by year of publication

Authors	Year	AD	Neuronal signals	ML	LC	#MLA
Sweeney et al. [17]	2012	✓	EEG	✕	✓	0
Khatwani et al. [18]	2013	✕	EEG	✕	✕	0
Barua et al. [19]	2014	✓	EEG	✓	✓	34
Rahman et al. [20]	2015	✓	EEG	✓	✕	1
Urigu¨en et al. [16]	2015	✓	EEG	✕	✕	0
Tandle et al. [12]	2015	✓	EEG	✕	✕	0
Islam et al. [21]	2016	✕	EEG	✓	✓	12
Jung et al. [22]	2016	✓	EEG	✕	✕	0
Lai et al. [23]	2018	✕	EEG	✕	✕	0
Manaan et al. [24]	2018	✓	EEG	✕	✓	0
Jiang et al. [15]	2019	✓	EEG	✕	✕	0
Sadiya et al. [25]	2021	✕	EEG	✓	✓	13
Fabietti et al.	2022	✓	EEG, MEG, ECoG, LFP, Spikes	✓	✓	127

*AD* artefact description, *ML* machine learning methods, *LC* literature comparison, *#MLA* number of machine learning articles compared

To address these challenges and facilitate access to appropriate ML and data-driven artefact detection and removal method, we developed ABOT (Artefact removal Benchmarking Online Tool). The literature was surveyed as resources for the tool to create an up-to-date dataset and define key features for users to compare. A review of the different approaches was carried out to complete a more thorough report. Therefore, this paper provides the following contributions in the area of artefact detection and removal in neuronal signals:

- Creation of an online tool for the neuroscience community to use, also available through an open-access repository.
- Compilation of a comprehensive bibliographic dataset of ML-based methods for artefact detection and removal from neuronal signals and defining and extracting key features for comparison.
- Reviewing the methodologies across all signal modalities.

This article is divided into seven sections. In Sect. 2, the different signal acquisition methods are presented, followed by the details of the different possible artefacts. Section 3 describes the online benchmarking tool from the software development perspective. Subsequently, Sect. 4 covers the creation of the bibliographic dataset, continued by the review of the collected articles in Sect. 5. Lastly, Sect. 6 discusses challenges and future perspectives within the field, and Sect. 7 makes concluding remarks.

## Neuronal signal acquisition

**Fig. 2:**
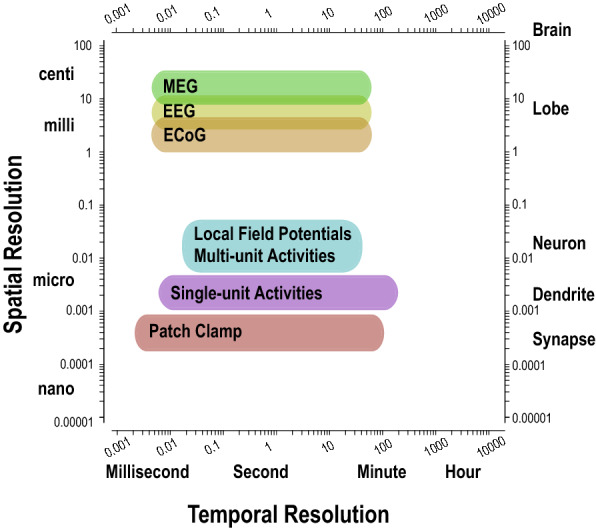
Spatio-temporal resolution of EEG, MEG, ECoG, LFP, multi-unit activities, single-unit activities and patch-clamp recordings. Modified from [23]

There are distinct neuronal signals depending on the recording techniques, i.e., invasive or non-invasive. The non-invasive type includes those obtained by EEG as well as MEG. In contrast, the invasive type includes ECoG, LFP and neuronal spikes (including multi-unit activities, single-unit activities and patch-clamp recordings). The spatiotemporal resolutions of these techniques are shown in Fig. 2.

### Non-invasive signals

The principle behind MEG is acquiring the magnetic fields generated by the electric currents circulating in the neurons. These fields are 10−14 Tesla for evoked fields to 10−12 Tesla in an epileptic episode and can be detected in cortical and subcortical regions by modern systems. These signals are captured by very sensitive magnetometers, called SQUID (superconducting quantum interference device), that require to be cooled at near absolute zero temperatures. Due to their sensitivity, the device must be stored in a magnetically shielded room to avoid external interferences. The advantages of the methods are that the spatial resolution of MEG ranges in a few millimetres, and its temporal resolution is less than a millisecond, fast enough to detect any neuronal process. Furthermore, magnetic signals are much less dependent on the conductivity of the extracellular space. Thus, skin and scalp muscles do not cause disturbances [30]. However, MEG must be used in combination with magnetic resonance data in order to create activation maps, as it does not provide anatomical information.

EEG records the electrical impulses coursing through the excitation of dendrites of several pyramidal neurons in the cerebral cortex. The EEG signal acquisition, though depending on the application, usually records for 20–30 min and uses several different electrode layouts, with the most popular one being the 10–20 system, whose spatial resolution is in the cm range. However, several acquisition systems have recently been proposed to cover subcentimeter spatial resolution [31]. The electrodes utilised can be of different types: wet (requiring electrolytic gel or saline solution), semi-dry (tap water humidity) or dry (conductive foams, spring-loaded fingers), where the latter can be beneficial in long term-experiments as they don’t dry up, but have higher impedance and are more sensible to artefacts. The low-frequency range EEG works on can be assorted into Delta waves (0–3.5 Hz), Theta waves (4–7 Hz), Alpha waves (8–13 Hz), Beta waves (14–40 Hz) and Gamma waves (40 Hz). Since it possesses a low signal-to-noise ratio, elaborate processing is required to extract useful information [22]. The main benefits of EEG is that its portability allows to study real-time neuronal activity outside of laboratory settings, is non-invasive and requires a less complicated set-up, and modern technology advances had led it to be relatively inexpensive.

### Invasive signals

ECoG signals are obtained with electrodes placed in the epidural or subdural layers of the brain, making it an invasive procedure. Still, it bypasses the distortions produced by the skull and intermediate tissue. The first location has a spatial resolution of 1.4 mm, while the second one of 1.25 mm. As the signal acquired has more amplitude than EEG, it is less sensitive to the artefact. Depending on the size of the electrodes, it can be classified as conventional ECoG for larger areas (e.g., mm in diam.) or *µ*ECoG for small areas (e.g., 100 µm in diam.). Furthermore, this method possesses a broad bandwidth (0–500 Hz) [32]. During the recording direct cortical electrical stimulation is frequently performed with it in order to do a cortical functional mapping and identification of critical cortical structures. A limitation this method has is the limited time window for recording, so sudden events such as seizures may not be recorded during it.

LFPs are recorded by micro-electrodes (glass micropipettes, metal or silicon electrodes) in deeper brain layers by low-pass filtering of the extracellular electrical potential to under 100–300 Hz. The obtained signals encompass neuronal processes such as afterpotentials of somatodendritic spikes, synchronised synaptic potentials, and voltage-gated membrane oscillations. Given its ability to capture different activities within a wide range of frequencies, it indicates the contribution of several different neuronal processing pathways [33]. They are recorded both in vivo and in vitro (brain slices), and can be utilised for closed-loop neuromodulation.

The single-unit recording detects neuronal activity with a microelectrode, which requires its size to be in the order of micrometres. The measured action potentials' amplitudes are generally 0.1 mV and are sampled at a 1 kHz or above frequency. Electrical currents can be injected into the cell to change the membrane potential in order to learn about its conductance, referred to as voltage clamp, and it can be carried out via different configurations if the electrode is inside, outside or attached to the cell. When studying individual ion channels, a patch-clamp electrode is employed. If the electrode size is larger, it records the activities of a group of neurons, which is called multi-unit recording. Multi-unit recording can be used to distinguish the number of cells surrounding it, and which cell is the source for the spike is referred to as spike sorting [34].

### Artefacts

Each type of artefact manifest in a specific frequency, and amplitude bands can be periodic or irregular and single or multichannel. They have distinct sources, classified as internal (or physiological) and external (or non-physiological). We describe them in the subsequent paragraphs, but readers are directed to Ref. [12, 17] for more in-depth reviews.

The electrical activity within the body causes internal artefacts. The main ones include electrooculogram (EOG) generated by eye movements, electroretinogram and blinking [35, 36], electromyogram (EMG) produced by the contractions of muscles, electrocardiogram (ECG) caused by the electric activity of the heart [37] or the spiking activity of local neurons in extracellular recordings [38], in other words, spike leakage. In addition, there are other artefacts that are barely mentioned in the literature that we have surveyed, such as skin potentials or respiration [39–41]. External artefacts are those generated by electronic devices or external electromagnetic waves, e.g., power lines [42], cell phone signals and light stimulation [43]. In studies where electrical stimulation is done to the brain, said signal may also appear in the recordings, known as a stimulation artefact [10]. Lastly, artefacts may be generated due to instrumental errors in the recording process, such as an electrode's poor contact, popping, lead movements and electrode drift, i.e., changes in the electrode's position in relation to the brain [44].

In regard to their properties, they can be described by their frequency band and their shape. EOG changes the potentials of the electrodes in the frontal region, appearing as high amplitude 3–10 Hz signals, and its repetition produces slow waves similar to delta waves. EMG amplitudes and waveforms vary on the muscle and the degree of contraction, spanning a frequency range from 2 up to 200 Hz, and it can be harder to detect due to their fewer repetition than the other artefacts. ECG has a regular pattern with frequencies near 1.2 Hz, and an amplitude in the millivolt range. Respiration artefacts manifest in the 5–10 Hz range, overlapping with the theta band in rats. Interference artefacts such as the transmission line (50/60 Hz) can be easily removed using a notch filter, whereas cell phone signals are in the order of megahertz and can be avoided in the experimental set-up. Lastly, instrumental artefacts generated by poor electrode contact are of low frequency, whereas lead motions have a more irregular shape that bears little resemblance to neuronal activity.

Therefore, each artefact manifest in particular frequency bands, amplitudes and shapes, many that overlap with the neuronal signals of interest and even among themselves. Due to this, filtering without producing information loss or a distortion of the signal becomes difficult. As an alternative, computational methods such as ML techniques have been developed to identify and remove them automatically. Having reviewed the different neural signals and artefacts, the functionalities of the online tool are described in the following section.

## Online benchmarking tool

**Fig. 3:**
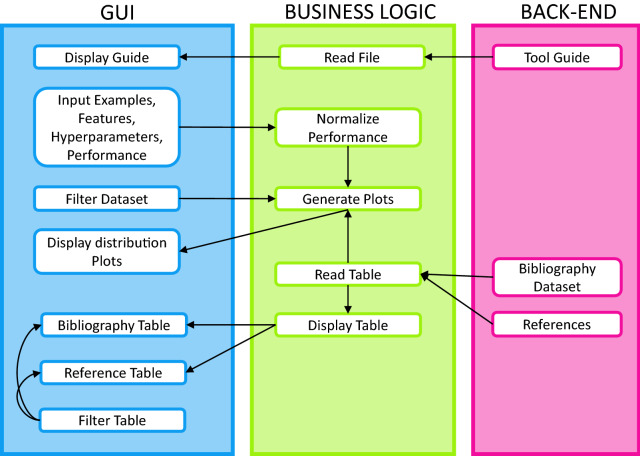
Functional block diagram of the online tool, where the relationships between elements of the graphical user interface, the business logic and the back-end are displayed

The online tool^1^ has been developed using the R language. It is dependent on the packages "Shiny" [81], which facilitates the construction of interactive web apps, "DT", which provides an interface to the JavaScript library DataTables, "shinyjs" for performing common useful JavaScript operations in Shiny, "shinyWidgets" to control the appearance, "shinyalert" for error messages, "readxl" to import the tables, "dplyr" to filter them, "ggplot" and "plotly" to generate the graphs. A user-friendly three-tab layout constitutes the app with a simplistic theme. A functional block diagram is displayed in Fig. 3. The relationships between the displays and user inputs in the GUI, the functions in the business logic layer, and the files in the back end are related. Upon opening the tool, a pop-up message is displayed introducing the app and how to operate it.

**Fig. 4:**
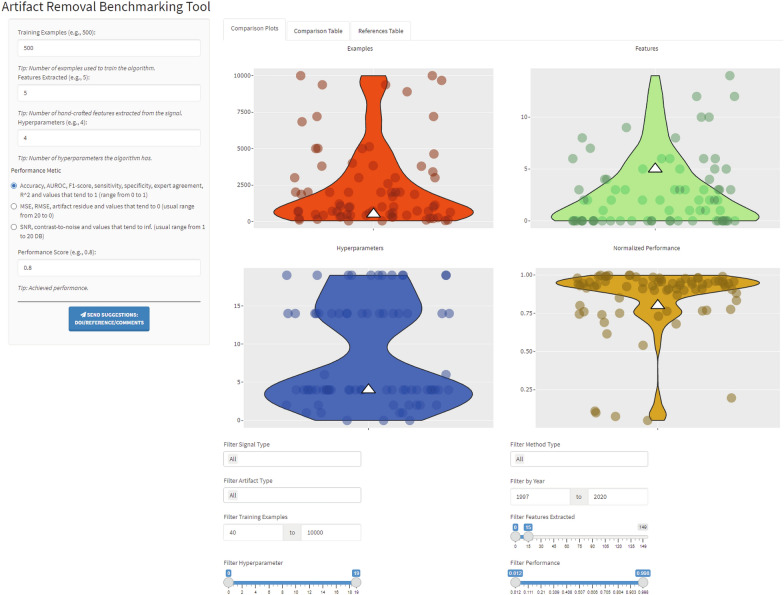
Screenshot of the main page of the online tool, where input values and display filters have been applied to showcase the functionality

A screenshot of the main page of the online tool is shown in Fig. 4. The user can input the metrics of their approach in the side panel (training examples, features extracted, hyperparameters, select which performance metric they have used and its value) to compare their results to the literature. In addition, they can send suggestions of literature to add or comments about the tool, such as features they wish to see implemented via an online form. The first tab, "Comparison Plots", has four plots, each displaying the violin plot and the scatter points of each metric from the collected dataset. Below them, options are available to filter the plots based on the signal, method or artefact type, year of publication, number of examples, features extracted, hyperparameters and normalised performance. Multiple filters can be used simultaneously, and if the search yields no result, an alert appears to notify the user. In addition, the app takes the inputs of the side panel and displays the value as a white triangle to differentiate it clearly from the other values. By hovering over a point, the collected information (reference, year, signal, etc.) specific to it is displayed, allowing easier identification for the posterior use of the other tabs.

**Fig. 5:**
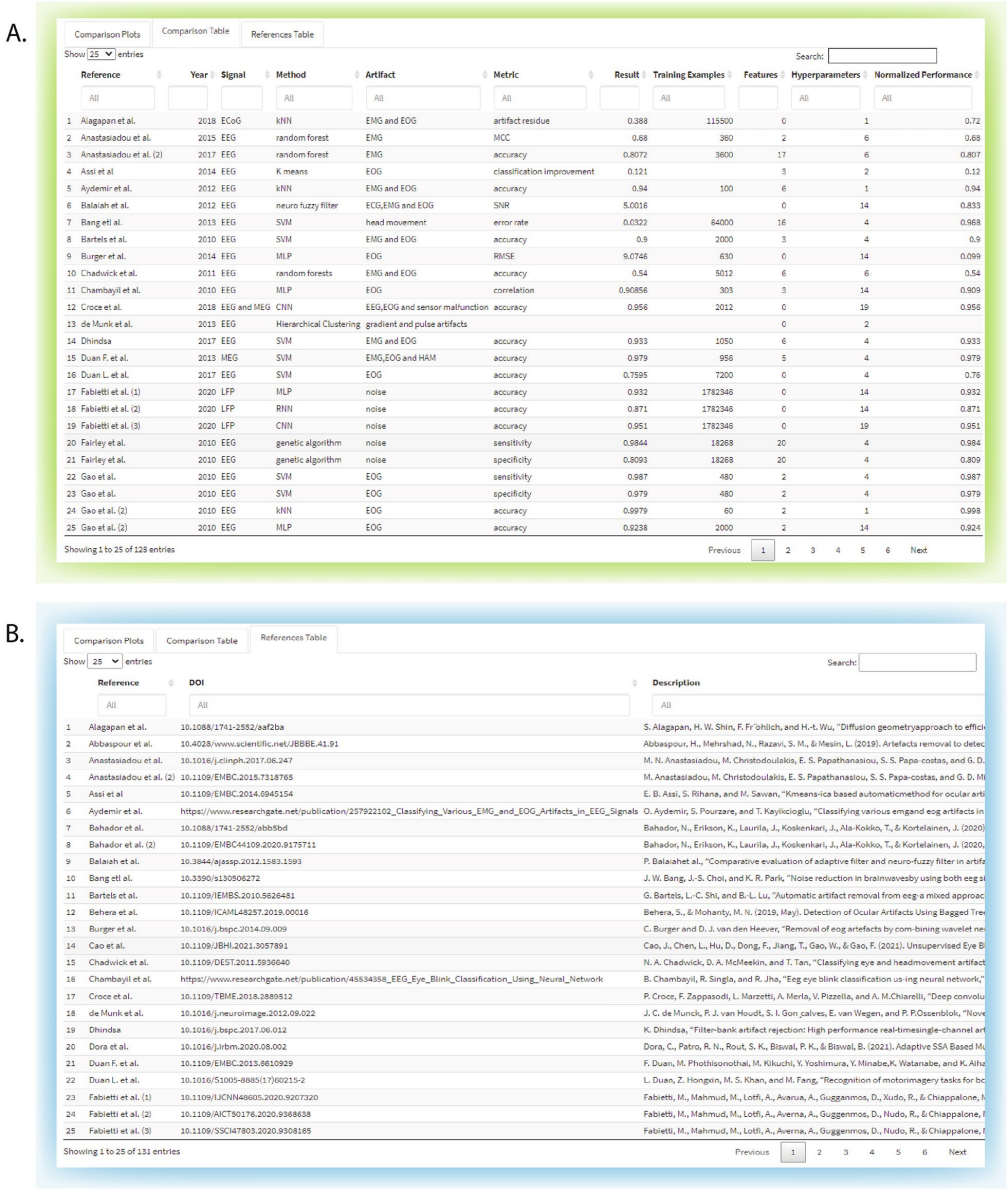
Second and third tab of the online tool, showing **A** the comparison table and **B** the references table

The second tab, "Comparison Table", contains the table with the methodologies found across the literature and their previously reported details, the original performance metric reported, and the normalisation used. The user can select how many entries are shown, filter through each detail, define intervals in numeric details, or use keywords to search for specific ones. The last tab, "References Table", contains the list of Digital Object Identifiers (DOI) and the complete references, which can be sorted by author or keywords as well. The second and third tabs are depicted in Fig. 5A and B, respectively.

The collected data and the tool's code are made publicly available^2^ to foster reproducibility by making it FAIR (Findable, Accessible, Interoperable and Reusable). This allows users to find relevant, multiple research models or customise one to their needs. In the next section, the creation of the tool's dataset from articles with ML methods for artefact removal and detection is presented.

## Bibliographic dataset creation

**Fig. 6:**
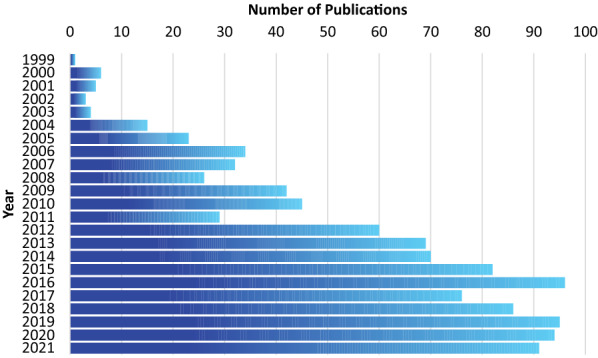
Publication trend in artefact removal from neuronal signals. The results were obtained by searching scientific databases with the term "artefact removal"

To survey the literature, three databases were searched: the ISI. Web of Knowledge database of the Clarivate Analytics,^3^ the IEEEXplore^4^ and the Scopus database of the Elsevier.^5^ The article title, abstract and author keywords fields of these three databases were searched using search phrases composed of "artefact removal" in conjunction with "EEG", "MEG", "ECoG", "LFP", and "spikes". The obtained results from three databases formed three datasets and were saved in separate comma-separated values (CSV) files. The datasets were compared and pruned by removing duplicate and irrelevant entries returned by the search results. The pruned and combined final dataset contained a total of 1084 publications whose yearly publication frequency is reported in Fig. 6.

Out of the shortlisted 1084 papers, manual scrutiny revealed that only 95 of them applied ML-based techniques in artefact detection and removal. To have the most up-to-date review, the aforementioned literature search is complemented with Google Scholar, reaching a total of 127 articles that apply machine learning to artefact detection and removal from neuronal signals.

**Fig. 7:**
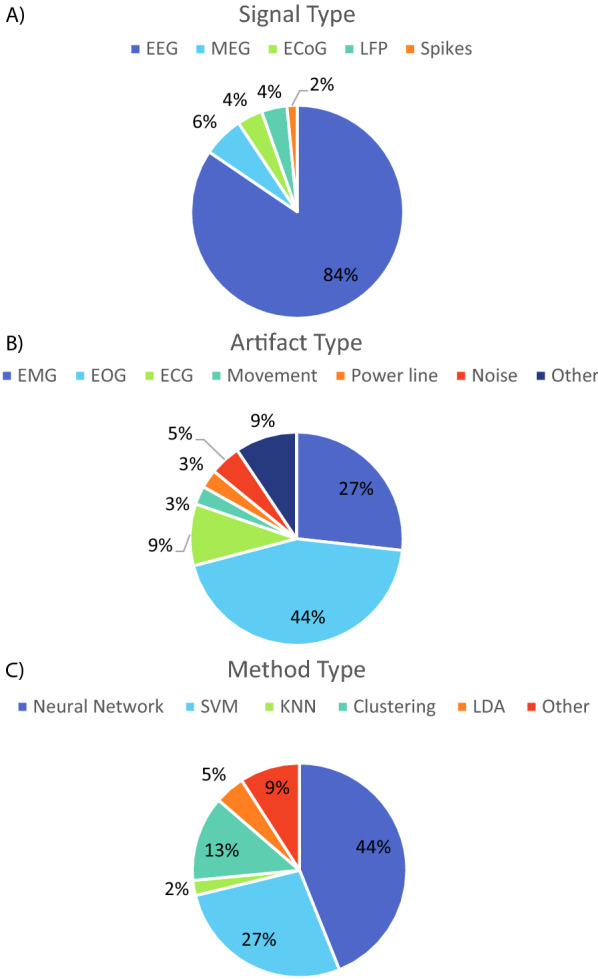
Distribution of the **A** signal type and **B** artefact type, and **C** method type in the extended dataset. *EEG* Electroencephalogram, *MEG* magnetoencephalogram, *ECoG* electrocorticogram, *LFP* local field potentials, *EMG* electromyogram, *EOG* electrooculogram, *ECG* electrocardiogram, *SVM* support vector machines, *kNN* k-nearest neighbours, clustering, *LDA* linear discriminant analysis and other

The distribution of the type of signal, artefact type and method type in the extended dataset is presented in Fig. 7. For each distribution, articles that dealt with more than one type had each of them counted separately. For example, an approach for EEG that deals with EOG and EMG using a single support vector machine (SVM) model equals values of: 1 EEG, 1 EOG, 1 EMG and 1 SVM.

There is a significant difference in the number of approaches published for EEG (84%), which is followed by MEG at 6% and the invasive recordings between 4 and 2%. This difference can be attributed to the accessibility of non-invasive recording methods and the number of open-access datasets [45]. Regarding the type of artefact, EOG and EMG represent 71% of all approaches. The former has high amplitude and disturbs mainly the recordings anterior scalp regions. In contrast, the latter has a large frequency span and the activity of the head, face and neck muscles are conducted through the entire scalp, so detecting and removing them is of vital importance. The remaining 29% is divided between ECG, power line, noise and others. The "noise" category was assigned to those which did not address the origin of the artefacts and referred to them as such, while the "other" included ones such as electrode pop, ballistocardiogram or electromagnetic interference. The most popular method has been neural networks (NNs) (44%), which are composed of multiple layers of neurons for processing non-linear information and were inspired by how the human brain works and are known to achieve good performances across domains, but require large amounts of information. They are followed by support vector machines (27%) and any form of clustering (13%), while the least applied are linear discriminant analysis (LDA) (5%), k-nearest neighbours (kNN) (2%) and other techniques (9%), which includes approaches such as swarm algorithms or sparse representation.

**Fig. 8:**
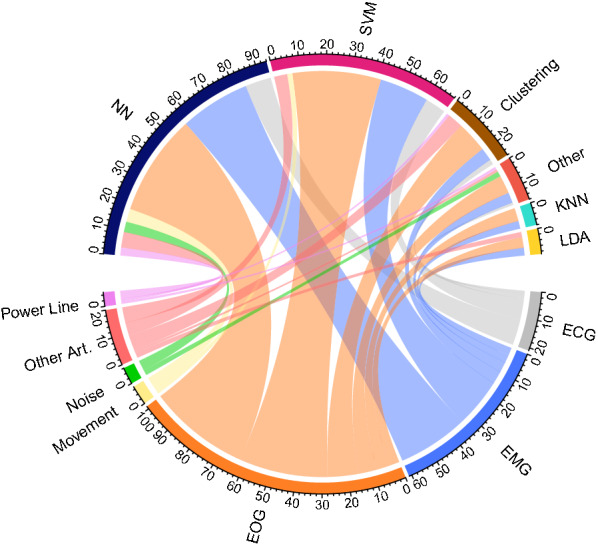
Frequency of artefacts and the used machine learning methods

In Fig. 8, the relationship between artefact type and ML method used to identify or remove it is shown. Both EMG and EOG follow SVM, NNs, clustering, and kNN, while ECG, noise, movement, power line and other artefacts are addressed mostly by NNs instead of SVM.

Our survey results show that there is no standard performance metric, constituting a challenge to compare the different results obtained by authors. To address this issue, Valipour et al. [46] have compiled the different metrics frequently used in research papers to consider the effectiveness of EOG removal algorithms. In addition, Islam et al. [21] stated the necessity for using a unique standard evaluation method composed of quantitative and qualitative metrics.

While developing a metric or scale suitable for all the different applications may not be feasible, to compare them, observing four key characteristics of ML models are proposed: the amount of training examples, the amount of extracted features, the model's hyperparameters and the model's performance. Machine learning models require information to make predictions accurately. However, the amount varies depending on each one. In particular, neural networks are known as data-hungry algorithms due to the amount they need to be trained; however, alternatives such as pre-trained models and one-shot learning help with this issue. Out of two models of equal or similar performance, the one which needs fewer data should be favoured. The second characteristic is the number of handcrafted features extracted. To choose handcrafted features that require expertise and represent the information fed to a model, one should penalise the number extracted from a signal. The third characteristic is the algorithm's number of hyperparameters (see Table 2). This was chosen to reward algorithms that are less complex to train.

**Table 2:** Examples of hyperparameters of machine learning methods

Method	Hyperparameters
Neural network	Learning rate Momentum Weight decay Epochs Batch size Number of hidden layers Neurons per hidden layer Neuron's activation function Regularisation Dropout Weight and bias initiation loss function Output function number of classes [47]
Convolutional neural network (spatial feature learning)	Patch size Convolutional layers Fully connected layers Number of filters Filter size [48]
Support vector machine	Kernel Cost Gamma Degree [49]
k-nearest neighbour	K
Linear discriminant analysis	None
Clustering	N clusters Distance function

The last characteristic is a normalised performance score, as described in the following expression:

1 $$\mathrm{Normalised performance}=\left\{\begin{array}{c}\frac{\mathrm{metric}}{\mathrm{max\,metric}}, {\text{if\,best\,metric}}=1\\ \frac{\mathrm{metric}}{1+\mathrm{metric}}, {\text{if\,best\,metric}}=0\\ 1-\frac{\mathrm{metric}}{1+\mathrm{metric}}, {\text{if\,best\,metric}}=\infty \end{array}\right\}$$

Here, the first case applies to metrics such as accuracy, the area under the receiver operating characteristic (ROC), F1-score, sensitivity, specificity, expert agreement, R2; the second one to mean squared error (MSE), root mean squared error (RMSE), artefact residue; and the third one to signal-to-noise ratio, contrast-to-noise ratio and others. Due to the lack of a standard metric for evaluating artefact detection and removal, we have devised Eq. 1 as a way of aiding the visual selection of methods, as the normalised performance scales all metrics between 0 and 1. This information is meant to be utilised in conjunction with the rest of the information provided in the tool via hoovering or via the description table, not as an absolute comparison criterion on the performance of the method. This proposed scale is the first step to closing a major gap in the field, and its limitations are discussed in Sect. 6.

**Fig. 9:**
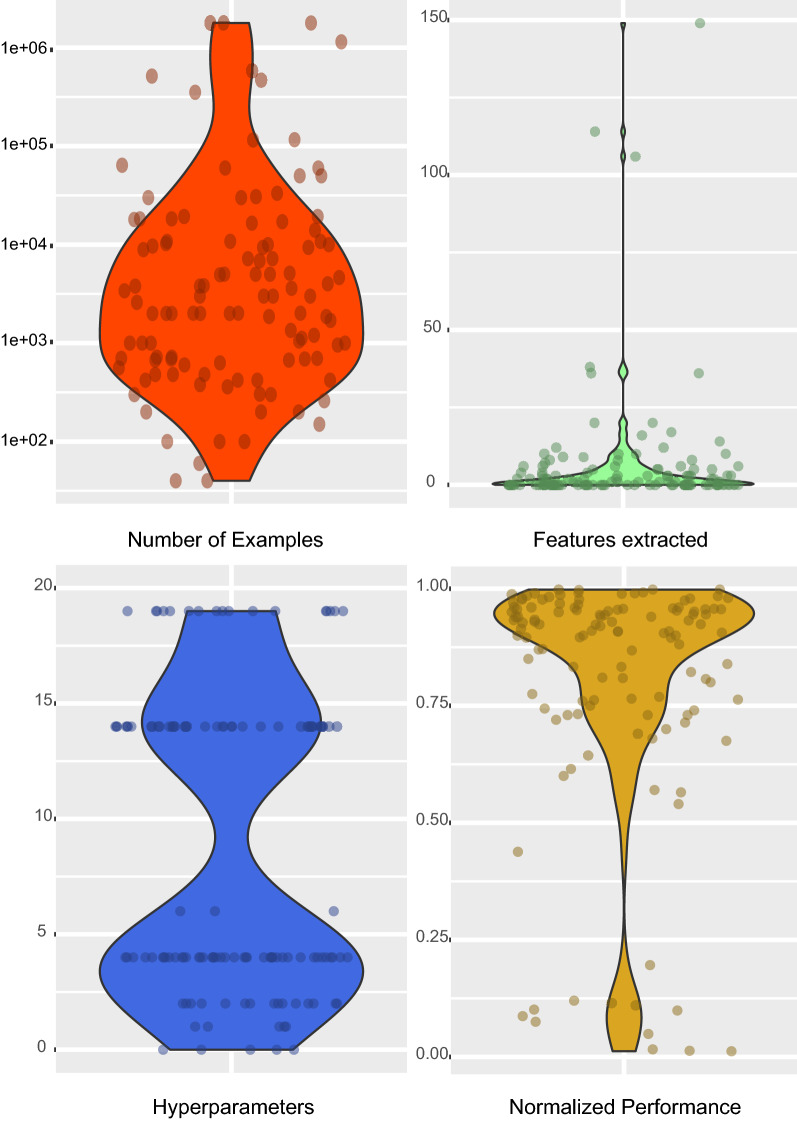
Distribution of the four characteristics from all the collected articles

These four characteristics were chosen because they are the most consistently reported elements that can be used to compare the algorithms. In contrast, time complexity, memory complexity, parallelisability, portability and interpretability were inconsistently reported. The selected characteristics were extracted from all the collected articles, and their distributions are displayed in Fig. 9. Overall, the majority have used less than 65,000 training examples, which is expected for machine learning models. Regarding the features extracted, most of them are under 20 with the presence of some outliers. As neural networks do not depend on them to achieve good performance and are the most utilised technique (see Fig. 7), the distribution is right-skewed. The hyperparameters take discrete values, generating a gap in the distribution where there are no methods with that particular amount. The upper half is the neural network approaches, which present 44% of the total approaches, and the lower half is the remaining methods. Lastly, the normalised performance concentrates above 0.7 since research is sought to be published after achieving a successful performance level. Values lower than that are due to the normalisation function, where removal techniques that haven't achieved low MSE are significantly penalised. Subsequently, we review the articles compiled in the dataset for a more in-depth analysis.

## State-of-the-art in artefact detection and removal

**Fig. 10:**
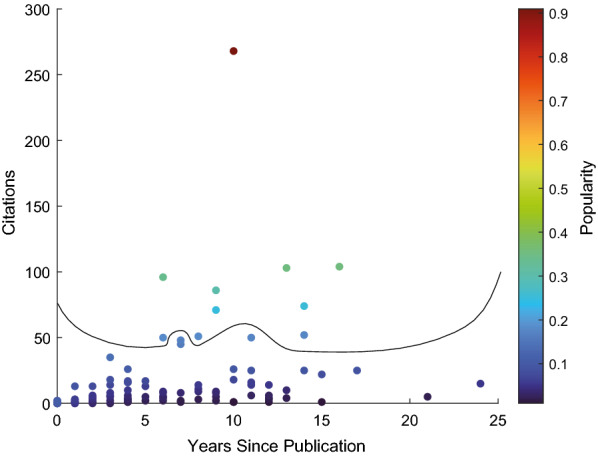
Popularity electroencephalography publications in relation to citations and years since publications, with the threshold line for the top 10 articles

In the following sections, the articles from the dataset are reviewed, organised progressively by each level of the acquisition method's spatial resolution. Given the extensive literature available on EEG signals, the most popular articles are chosen to be discussed, while the rest will be provided as a table in the online tool. We define popularity as the number of citations, however recentness also plays a factor in the number of citations, so we express the popularity of an article as stated in the following equation:

2 $$ {\text{Popularity}}_{i}  = \frac{{{\text{citations}}_{i} }}{{{\text{max}}({\text{citations}}_{{i, \ldots ,n}} )}}*0.9 + \frac{1}{{1 + {\text{years}}\,{\text{since}}\,{\text{publications}}}}*0.1, $$

where *i *= 1,…, *n* and *n* equal the number of EEG articles in the dataset. Figure 10 depicts the publication trend in artefact removal from neuronal signals as a function of the citations and years since publication. While the popularity index allows the identification of high-impact articles, it does have a negative bias toward more recently published articles that may be of high relevance. Nonetheless, the key information of those articles is available through the toolbox for users to explore and compare.

### Magnetoencephalography

Hasasneh et al. [50] developed an ECG and EOG artefact classifier based on a combined convolutional neural network (CNN), utilising temporal and spatial information of independent component analysis (ICA) components. From 48 subjects, 7122 examples were obtained after data augmentation, and the model achieved an accuracy of 94.4%. This has proven that accuracy improves when temporal and spatial information is incorporated. Additionally, the model was trained without relying on auxiliary signal recordings, and it allows for EEG and various sensor types as well.

Garg et al. [51] proposed two ECG and EOG identifiers composed of a deep 1-D CNN from ICA components. Resting state MEG data from 49 subjects were used to train the model and reached a 96% sensitivity and 99% specificity on the ECG model and 85% sensitivity and 97% specificity on the ECG model. Finally, gradient-weighted class activation maps were generated to visualise learned features, which shows how the model operates.

In another publication by Garg et al. [52], they applied a 10-layer CNN, which labels EOG artefacts. The MEG data were extracted from 44 subjects, out of which 14 were used for training and 30 for testing. The obtained accuracy on the testing set was 99.67%. The saliency maps and gradient-weighted class activation maps revealed that the model's learned features correspond to those used by human experts.

The approach by Phothisonothai et al. [53] consists of extracting the central moment of frequency, fractal dimension, Kurtosis, probability density and spectral entropy from independent components. Next, a Gaussian kernel SVM was trained to identify these features. From a dataset of ten healthy children, the obtained accuracies were 98.15%, 99.18%, and 92.33% for high amplitude changes (HAM), ECG and EOG, respectively.

Duan et al. [54] presented a weighted SVM as an ECG, EOC and sudden high amplitude change artefact predictor. This method was chosen to address the class imbalance factor of the independent components. By re-weighting, the examples belonging to the negative class, the specificity of the classifier was boosted. Using a dataset composed of the MEG data of ten healthy children, the model's accuracy was 97.91% ± 1.39%.

Rong et al. [55] applied two clustering methods to ICA components: threshold-based and an Adaptive Resonance Theory (ART) neural network. The characteristics compared for thresholding were the statistical aspects, topographic patterns, and power spectral patterns. The MEG data were acquired from five healthy right-handed adults, and the chosen performance metric was "correctness". This can be defined as the proportion of real artefactual independent components identified over the total independent components identified by the algorithm. The ART network achieved 60% correctness on ECG data and 70% on EOG data, underperforming considerably against the 100% and 90%, respectively, the threshold method achieved. Lastly, they compared the number of real artefactual independent components identified over the total artefact independent components in the dataset to measure named "coverage" to measure the underestimation of artefacts. This showed that the coverage of the network was approximately 85% over both artefacts.

Croce et al. [56] trained a CNN with the independent component's spectrum and the topographic distribution of its weights, extracted from multichannel MEG and EEG recordings. From 503 brain and 564 artefact components of the EEG recordings along with 2730 artefact and 2019 brain independent components of the MEG recordings, the final dataset was downsampled to 2012 (503 each category). The classification accuracies obtained through cross-validation were 92.4% for EEG, 95.4% for MEG and 95.6% for EEG + MEG.

Lastly, Treacher et al. [57] employed a combination of 1 dimensional CNN for the independent components and a 2-dimensional CNN for the spatial maps to detect eye blinks, saccades and cardiac artefacts. The data set was composed of 294 scans from 217 subjects, out of which 232 scans or 49,100 independent components were used to train the model. After hyperparameter optimisation of both networks, an accuracy of 98.87% was achieved on the test data by the ensemble model, surpassing the performance of the individual temporal and spatial models.

In the case of MEG, we can observe that researchers constructed both artefact-specific models and multiple-artefact models. The model by Duan et al. is able to identify multiple artefacts with near 98% accuracy, a performance comparable to the models of other authors that are able to identify a single artefact. Treacher et al. also identify multiple artefacts but is more computationally expensive as it requires training two CNN models, and the model developed by Croce et al. was trained jointly with the data of EEG, which may not be available in most experiments.

### Electroencephalography

Winkler et al. [58] proposed an ICA-based approach that estimates the source components for the classification of general artefacts by factoring in temporal correlations, named temporal decorrelation source separation. Components extracted from data of 12 healthy right-handed male subjects during two auditory stimuli in an oddball paradigm were labelled by two experts. They were broken into 690 examples for training and 1080 examples for testing a Linear Programming Machine, a Gaussian kernel SVM and a regularised LDA model. The LPM classifier obtained a classification error of 8.9% based on six handcrafted characteristics, while the difference between the two expert scores was 13.2%. For validation, they used data from two studies: 18 subjects in an auditory event-related potential paradigm and 80 subjects in the motor imagery BCI paradigm. The former dataset achieved an average MSE of 14.7%, compared to 10.6% disagreement between experts. At the same time, the latter showed that eliminating up to 60% of the framework did not affect the overall performance of the BCI classification.

Shao et al. [59] applied a weighted version of SVM to handle the inherently unbalanced nature of ICA's component classification. By giving a higher penalty on the classification errors generated by the minority class samples, the algorithm compensates for the bias of prior class probabilities. EEG recordings were obtained from ten right-handed volunteers, segmented into 12 s epochs and then decomposed each one into independent components by the ICA. Each independent component was manually labelled, and six features were extracted from them to train the models, trained with the recordings of 9 subjects and tested with the left-out subject. The compared models included the Gaussian mixture model, kNN, LDA, standard SVM and weighted SVM with and without error correction. The weighted SVM obtained the best results with error correction, an accuracy of 95.67%, and a reduction of 98.4% and 96.8% in the epochs of ECG artefacts and EOG artefacts, respectively.

Shoker et al. [60] used ICA with SVM with handcrafted features. Ten 7-min-long EEG data sets were built with data supplied by King's College Hospital, London, UK. After applying the blind source separation method, 200 independent components were obtained: 100 free of artefact and 100 containing eye blinks; from them, four handcrafted features were extracted and used to train the classifier. The SVM was trained using linear, cubic polynomial and Gaussian kernels, with the latter achieving the highest accuracy of 98.5 ± 1.00%.

Hader et al.'s [61] approach consisted of the application of ICA and SVM on the topography and power spectral density of the independent components. Four different artefacts were recorded from four healthy and paralysed subjects to train a Gaussian SVM using 20-fold cross-validation. The accuracy was 99.39% for eye blinks, 99.62% for eye movement, 92.26% for jaw muscle and 91.51% for forehead, averaging 95.70% between them.

Lawern et al. [62] addressed artefact removal by means of implementing auto-regressive models for feature extraction coupled with a Gaussian SVM classifier. Seven participants made a series of facial and head movements that induced artefacts, which involved moving the jaw vertically, clenching the jaw, moving eyes left, moving eyes upwards, blinking both eyes, moving the eyebrows and rotating the head. An eight multi-class SVM was trained with these recordings, using fourfold cross-validation to determine its optimal parameters, finally reaching a 94% accuracy.

Gao et al. [63] presented a method where ICA is applied to obtain independent components; then, a peak detection algorithm recognises eye-blink artefacts, followed by a classifier trained on the topographic and spectral features to recognise eye-movement artefacts and finally, the artefact-free components are used to restore the signal. Their dataset was composed of 600 EEG epochs from 15 healthy subjects for 3 s per epoch. They compared three different classifiers: MLP, Fisher Discriminant Analysis and SVM, with the latter achieving the best scores of 98.7% sensitivity and 97.9% specificity, using tenfold cross-validation.

Li et al. [64] employed the Lomb–Scargle periodogram to determine the spectral power from recordings that had parts contaminated by artefacts removed and used those features to train an autoencoder and a Gaussian SVM. Evaluated with simulated and real motor imagery data, the autoencoder proved to be comparable to the SVM. Moreover, results show that accuracy is not reduced dramatically if various amounts of data are discarded. Therefore, they concluded that rather than discarding an entire segment, it could use all the same to generate commands after removing the parts with artefacts.

O'Regan et al. [65] proposed complementing EEG signals with gyroscope signals to detect head-movement artefacts. They collected data on head movement from seven healthy male adults for 30 min. Both types of signals were preprocessed and divided into epochs for the analysis. A total of 69 and 80 features were extracted from each epoch of the EEG and gyroscope signals. For each type of signal, a Gaussian kernel SVM classifier was trained, and a third feature fusion classifier surpassed the former two. The fusion model reached an average AUROC of 0.822 for the participant independent model and 0.98 for the participant dependent model. This shows that additional information about the presence of EEG artefacts is given by gyroscope features and boosts their detection. Nguyen et al. [66] named "wavelet neural network" their EOG detection methodology. It is composed of three steps: (i) decompose the raw signal into a group of wavelet coefficients; (ii) pass the coefficients in low-frequency wavelet sub-bands to an MLP for correction; (iii) reconstruct an artefact-free signal based on the corrected coefficients. The technique was trained on simulated data and validated on two datasets, recorded during a visual selection task and a driving test. The authors achieved an RMSE of 12.2 for the driving dataset and 19.21 for the visual selection dataset, surpassing the results they obtained with ICA. Furthermore, the solution is computationally efficient and more practical than ICA, suggesting an online deployment is feasible.

Gonçalves et al. [67] focused on removing artefacts in EEG from the magnetic resonance sequence magnetic fields during the co-registration of EEG and functional magnetic resonance imaging. They utilised a hierarchical clustering algorithm, which employs Euclidean distances to aggregate the different pulse artefacts. The averages of each cluster were then used to generate an artefact template that was subtracted from the respective pulse occurrences belonging to each cluster. The artefact correction in this situation has no ground truth to compare the outcome of the correction algorithm. Nonetheless, the authors used the estimated acquisition time of one slice to determine the quality of the successful correction.

We can observe that most of these articles share the commonality that they have utilised SVM as the classification model. From them, Lawern et al. has been able to achieve a performance nearing 96% in a model that is able to identify 7 types of artefacts, the most out of any article reported in the literature. This is achieved with only a second-order auto-regressive model as a feature, and was tested in real patient data. A benefit of the feature is that it is scale-invariant, so it is stable across subjects and computationally efficient to calculate, in contrast to ICA-based approaches. However, Lawern et al.’s approach must be used in conjunction with other methods for those looking to recover the underlying signal.

### Electrocorticography

Alagapan et al. [68] developed an artefact removal algorithm for ECoG labelled shape adaptive non-local artefact removal (SANAR). This approach works by approximating the Euclidean median of k-nearest neighbours of each artefact in a non-local manner, acquiring a template of the artefact, which then is removed from the original signal. It was applied to data obtained from a single subject carrying out a working memory task while being simultaneously stimulated, as well as a simulated ECoG and direct cortical stimulation, where an antenna connected to a function generator acts as a virtual dipole, and a saline solution emulates the conductivity of the grey matter. Artefact residue index was used to measure performance, which should be close to 0. ICA achieved 0.430 ± 0.015, while SANAR 0.388 ± 0.011, reaching better performance. Nonetheless, one must consider the extended calculation time as one of the main limitations of the method.

From another perspective, Tuyisenge et al. [69] developed a model for detecting bad channels in ECoG recordings of seizure patients undergoing pre-surgical recordings and stimulation. They extracted the correlation, variance, deviation, amplitude, gradient, Hurst exponent and Kurtosis from each channel and fed it to a bagging tree model for classification. They explored the model's performance based on the number of subjects used to train it, which plateaued at 99.7% accuracy with 110 subjects. The wrong channels consisted of artefacts such as electrode pop, power line noise and intermittent electrical connection.

Nejedly et al. [70] proposed using CNN with five different frequency bands of the recordings as inputs to identify between physiological, pathological, noise and muscle activity and power line noise. Their analysis was made using two large datasets. They made a general model (trained with one dataset and validated with the other) and a specific model (retraining the general model with 8% of the second dataset and validating with the remaining data). The general model achieved an F1-score of 0.89 in the noise and muscle activity class, while the specific model achieved 0.98 and 0.97 in power line and noise and muscle activity classes, respectively. The overall performance of the specific model was 0.96, including the physiological and pathological ECoG classes.

Finally, Fabietti et al. [76] explored the impact of sampling frequency in the four-way classification of baseline brain activity, seizure, line noise and noise and muscle activity. After down-sampling to balance the classes; they used 67,992 examples to train a CNN. At 5 kHz, they achieved a sensitivity of 99.7% for the line noise class and 91.9% for the artefact class. When the signals are downsampled to 250 Hz, the respective sensitivities are 99.4% and 87.8%, indicating a small loss of performance for a sequence reduction of 20 times.

Taking these articles into consideration, Tuyisenge et al.’s approach to utilise bagged decision trees achieves the best performance of artefact detection in ECoG signals. The performance was tested on the left-out data of 100 patients, indicating the robustness of the method. It is also worth highlighting that they utilised the least amount of training examples, as it was not a deep learning model, and achieved the performance with only 7 handcrafted features.

### Local field potentials

Regarding artefact detection in LFP, Fabietti et al. have explored several approaches. Their dataset comprises multi-site electrode recording in freely moving male Long Evans rats. First [72], they proposed using a multi-layered perceptron for the binary classification of LFP and artefacts of various origins. They explored how the performance varied based on the input length in both subject-specific and cross-subject models. The cross-subject model achieved an accuracy of 93.2%. This was followed by their second work [68].

A recurrent architecture, namely a long-short term memory (LSTM), was also used for binary classification and an approach based on forecasting. After comparing different parameter combinations, the best classification model achieved an accuracy of 87.1%, while the forecasting approach could not identify the two classes with good performance. The third approach [73] consisted of using CNNs, where three popular architectures were adapted for the one-dimensional signal. The best performance was achieved by the Alexnet [74] inspired model, with an accuracy of 95.1%. In addition, grad cam maps were extracted to understand which portions of the signal the model used for assigning each class. Continuing to explore interpretability, a decision tree-based model was the basis for the fourth research article [75]. They explored three feature extraction toolboxes combined with three feature selection methods to obtain an accurate and interpretable model. The accuracy of the decision tree was 89.3%.

From the artefact removal perspective, Fabietti et al. [77] proposed using an LSTM network to forecast "normal" neural activity to replace the artefactual segment. An open-source dataset of rodents in a treadmill was used to train the model, fed 200 ms long sequences and was asked to predict the subsequent data point in a sliding window approach. The performance was evaluated as the RMSE of 100 ms across four individual subject models and a cross-subject model, which achieved a performance of 0.189 in the test set. Afterwards, the generated signals were compared in the temporal and spectral domains, where they mimic the properties of the physiological recordings. These approaches have been compiled into an open-access toolbox for artefact detection and removal [78].

In general, it can be said Fabietti et al. has compared a wide range of architectures for artefact detection in LFP, and over two datasets the CNN [74] has achieved the best accuracy and the lowest computational time to classify a minute of recording. In regard to artefact removal, the use of LSTM to forecast over corrupted LFP segments has shown promise, and may prove use useful in single-channel EEG applications.

### Spikes

Klempivr et al. [79] approached artefact detection using transfer learning with a CNN based on AlexNet. The dataset was composed of thousands of 10-s extracellular microelectrode recordings of 58 patients with Parkinson's disease. Approximately 75% of the recordings did not contain any artefacts, and the preprocessed dataset consisted of nearly 100,000 one-second signal segments. Continuous wavelet transform was applied to generate a time–frequency image, which was the input to the network. This pipeline attained an accuracy of 88.1% for artefact identification and 75.3% accuracy for the individual classes of artefact identification.

From another angle, Hosny et al. [80] explored the use of machine learning to detect artefacts from multi-electrode recordings. Their data consisted of recordings from 17 Parkinson's disease who showcased artefacts such as mechanical motion, electromagnetic interference, baseline drift, irritated neuron and others. Power spectral density and wavelet packet decomposition was used to obtain 106 features, which were used to train classifiers such as Gaussian SVM, decision trees, AdaBoost, Bagging learners, LogitBoost and an LSTM network with 3785 examples. The best performing model was the LSTM network, with an accuracy of 97.49% on the test set.

Overall, Hosny et al. out-performs Klepmvir et al. in regard to the achieved accuracy on binary classification. Furthermore, Hosny’s model was trained with nearly half the amount of examples that the latter used, and the examples included a wider range of artefacts. However, training a LSTM network end-to-end is significantly more computationally expensive than to apply transfer learning to the Alexnet CNN.

We proceed to discuss the challenges in the field and the advantages and limitations of the tool in the subsequent section.

## Discussion

The toolbox allows filtering the data to find approaches that match the application of interest and compare them. However, if the user does not filter the data, is it a valid comparison between different types of signals, types of subjects and types of artefacts? For example, a method for removing muscle artefacts from human EEG recordings may not be very useful when developing or searching for methods for removing stimulation artefacts in a rodent's patch-clamp data. The difference between the different neuronal signals across subjects is significant enough to expect deviations when adapting from one to another. In addition, preprocessing such as filtering and feature extraction may also be needed to be adapted as means to obtain a working model. Regarding adopting a model of one type of artefact to another, some authors have applied the same model to different artefacts and achieved similar performances [52, 82, 83].

Furthermore, the approaches that address multiple types with a single model do so at a performance similar to other approaches with the same model that deal with only one type. Overall, the comparison outside the application must be done diligently, knowing that it does not mean that the performances will be maintained. Still, it can help orient those looking for which method to apply by discarding those that don't perform well. The normalised scale may also miss-represent results; for example, a high classification accuracy will be mapped to a value near 1; however, one must achieve an extremely low RMSE to achieve the same results. Nonetheless, it provides an approximation of the performance of the approach, which can be evaluated further on the table with the same metrics if desired. To the best of our knowledge, no other attempts to solve this issue have been made before so that it can be used as a starting point.

Out of the many challenges the field currently poses, replicability is the main one. Most studies have used private datasets, and outside the few hosted in physionet [84] or BCI competitions [85], the data have been removed from their respective websites. A limitation of this work is that despite the key characteristics of the approaches have been listed, details such as preprocessing steps or the layers of the neural networks are not listed. That information can be behind paywalls, leading to the inability to reproduce and compare results among studies. A second shortcoming is that the listed characteristics may not be sufficient for some researchers to decide; for example, the processing time is crucial information for selecting algorithms when looking for online implementations. However, the selected characteristics mainly were present throughout the surveyed literature, whereas others, such as the normalisation procedures, hyperparameter values, hardware utilised for training and computational time, were very inconsistent. Lastly, the toolbox focuses on machine learning solutions, excluding the wide range of artefact removal methods listed in Sect. 1. While this limits the tool's utility for those looking for the "best approach across the board", it is hoped that it will be useful for those looking at data-driven solutions or those with academic purposes such as method development or comparisons of machine learning methods.

Moving forward, automatic removal has a significant role to play in neuroscience. Craik et al.'s [86] review of deep learning for electroencephalogram classification tasks indicates that 63% of the studies did not methodically remove the artefact. Moreover, 29% removed them manually, and only 8% of studies used automatic methods, which mainly relied on ICA-based algorithms. In addition, Roy et al.’s [87] deep learning-based electroencephalography analysis review showcases in their survey that 47% did not remove artefacts, 30% did not mention if it was applied at all, and only 23% removed artefacts. Thus, there is an excellent opportunity to apply the methods listed in the tool in classification and other tasks. As previously indicated in Fig. 6, there is a consistent growth in the number of articles published every year that mention artefact removal. Filtering them, extracting the key characteristics and incorporating them into the tool takes time, another limitation of the proposed work. However, we hope that users will help us with its improvement via the suggestion email option by drawing our attention to where updates are needed. Hopefully, this will mean that the tool will remain valuable and necessary.

As online processing is taking more relevance, computationally and energy-efficient methods are desired. The trend shows that machine learning will most likely be the future direction in the field, given that those approaches suit the requirements mentioned above. This means that the next step is focusing on developing more interpretable models, especially those that include neural networks, providing insight into how variables interact and the model operates. In addition, models should allow interaction, such as choosing which artefact to detect, turning it off when it is not required, or allowing modification of the classification probability threshold. There is no wide range of techniques that excel for all possible artefacts and conditions; therefore, approaches should improve the robustness across multiple subjects and different biological contexts [88]. The use of several processing stages in which each phase eliminates each artefact to increase the signal's quality by using a series of algorithms remains a possibility [16].

## Conclusion

To analyse brain signals without interference from artefacts, researchers have proposed different means to detect and remove them. Because of the extensive literature on the topic and the wide range of signals, artefacts, and ML techniques, we have developed an online tool that facilitates browsing through the literature. The user can compare the performance of approaches for benchmarking or for implementation via the graphs and tables available in the tool. We have successfully surveyed the literature and extracted key characteristics of the different machine learning methods for the tool to showcase. In addition, the compiled articles were reviewed for a more comprehensive analysis. We hope the community adopts the tool; for that purpose, we have made it open-access and made its code available and allowed users to send suggestions via the tool. By facilitating the benchmarking of new methods, as the state of the art of artefact detection and removal techniques improve over time, so will the results of brain studies and BCI applications.

## Data Availability

The source code of the toolbox is available at https://doi.org/10.5281/zenodo.5789913.

## Footnotes

1 https://nachodev.shinyapps.io/ABOT/.

2 https://doi.org/10.5281/zenodo.5789913.

3 https://apps.webofknowledge.com/.

4 https://ieeexplore.ieee.org/.

5 https://scopus.com/.
